# How material sensory properties and individual differences influence the haptic aesthetic appeal of visually presented stimuli

**DOI:** 10.1038/s41598-024-63925-9

**Published:** 2024-06-13

**Authors:** Marella Campagna, Rebecca Chamberlain

**Affiliations:** 1 Department of General Psychology and Methodology, University of Bamberg, Bamberg, Bavaria Germany; 2 Department of Psychology of the Arts, Neuroaesthetics and Creativity, Goldsmiths University of London, London, UK

**Keywords:** Human behaviour, Sensory processing

## Abstract

Touch plays a crucial role for humans. Despite its centrality in sensory experiences, the field of haptic aesthetics is underexplored. So far, existing research has revealed that preferences in the haptic domain are related to stimulus properties and the Gestalt laws of grouping. Additionally, haptic aesthetics is influenced by top-down processes, e.g., stimulus familiarity, and is likely to be modulated by personality and expertise. To further our understanding of these influences on haptic aesthetic appraisal, the current study investigated the imagined haptic aesthetic appeal of visually presented material surfaces, considering the role of haptic expertise, Need for touch, personality traits. The results revealed a positive influence of familiarity, simplicity, smoothness, warmth, lightness, dryness, slipperiness and a negative influence of complexity on individuals' aesthetic responses. While the study failed to support the predicted influence of Need for touch and haptic expertise on aesthetic responses, results did reveal an influence of openness to experience, conscientiousness and neuroticism. Despite the limitations related to the indirect stimuli presentation (vision only), the findings contribute to the relatively unexplored role of bottom-up and top-down features in haptic aesthetics that might be incorporated into the design of consumers’ products to better meet their preferences.

## Introduction

Touch is a crucial sensory modality and given its powerful affective and cognitive components it is an important conduit for pleasurable and rewarding experiences^1^. Our sense of touch emerges early in ontogeny^2,3^, is characterized by the widest bodily distribution of sensory receptors relative to any other sense^4^, and remains the last sense to deteriorate with the aging process^5^. Due to its reciprocal character—active (haptic) and passive (tactile)—touch is crucial for fostering social bonds through oxytocin^6,7^ release and maintaining psychological well-being via interpersonal stimulation^8,9^. This effect is mediated by C-tactile fibers, slow-conducting afferents located in hairy skin regions, which are specifically responsive to the affective dimensions of touch^10,11^.

For the present study purpose, we focused on the imagined “haptic” feel of materials, involving the “imagined” dynamic, interactive processes that combine sensing, influencing through touch, as opposed to the purely perceptual and passive imagined “tactile” feel.

Despite its centrality to survival, and its intrinsic capacity to stimulate arousal, little is known about the hedonic aspects of haptic stimulus processing.

So far, the field of haptic aesthetics has revealed some underlying preferences in relation to stimulus properties, which are shared by other sensory modalities. The Gestalt principles influencing perceptual grouping (e.g., similarity, proximity, closure) affects the perception of haptic stimuli in both unimodal, and cross-modal settings^12,13^ and individuals seem to prefer complex order (unity in variety) in the haptic domain^14^. Additionally, the occurrence of the *Aesthetic Aha phenomenon*^15^, whereby individuals experience a pleasurable sense of reward in perceptually challenging circumstances, also takes place in the haptic domain. It was observed that liking judgments were significantly associated with situations under which both interest (given by high complexity) and pleasantness (given by strength of insight) were high. However, it is worth noting that haptic pleasantness was also associated with the material feel. It can be inferred that when tactile aesthetic judgments are to be made, individuals favor an optimal balance between fluent perceptual processing, provided by a sense of the whole, and a certain level of complexity, fulfilling a sense of accomplishment, intrinsically linked to humans’ innate tendency to explore^16,17^.

Taking the properties of the stimulus into account, recent investigations in haptic aesthetics have shown that micro (texture) and macro (shape) geometric properties of materials predict aesthetic responses. Specifically, it was found that pleasantness ratings associated with materials were inversely related to their coefficient of friction^18^, thereby suggesting a general pattern for aesthetic pleasure to decrease with the intensity of the stimulus^19^. The general tendency for roughness to be perceived as less pleasant was empirically confirmed by several studies adopting both unimodal (touch only/vision only) and bimodal (vision and touch) conditions, as well as active and passive touch. In a similar vein, Etzi et al.^20^ found cross-modal correspondence for everyday materials (e.g., cotton, silk, sandpaper, tinfoil), where smooth, soft textures were systematically associated to fictitious word with round-shape sound, i.e., *maluma,* and positive labels, e.g*., beautiful*, *light, bright*.

It can be inferred that humans are biased towards certain haptic stimuli that elicit *s*afe and comforting sensations, which are driven by the stimulus properties themselves as well as through the experience of the individual. As noted by Klatzky and Peck^32^, Nagano et al.^33^, attractiveness to human touch, which constitutes an integral part of the haptic aesthetic experience, seems to depend upon the apparent comfort of materials—smooth, soft materials invite human touch more than bumpy ones. Furthermore, studies conducted on blind and sighted participants found that they preferred curved, round and symmetrical 3D objects over sharp ones^34^. The preference towards smooth, soft materials and round shape, can be potentially explained as an innate tendency to favor stimuli that evoke explicit/implicit positive memories (e.g., maternal touch).

The tendency to want to touch objects, however, is also mediated by top-down processes, individual differences. Effects of familiarity on aesthetic preferences which are known to have an impact in the visual, auditory domains, have been similarly reported for touch^21–23^. For example, Suzuki and Gyoba^24^ found a mere-exposure effect in cross-modal interactions between vision and touch; previewing 3D objects significantly increased participants’ haptic preference in later exposure. The presence of a haptic mere-exposure effect can account for differences in individual responses towards materials, highlighting the potential interplay of aesthetic responses and haptic experience in terms of manual dexterity and haptic expertise^21,22,25,26^. Across aesthetic domains experts do appear to perceive sensory stimuli in a different manner to non-experts, as a result of enhanced perceptual abilities, memory capacities, and exploration strategies^27–31^.

The role of personality traits in influencing sensory perception and aesthetic experience has also been widely demonstrated across other sensory modalities such as taste and smell^36–38^. For instance, research has highlighted a robust link among personality characteristics and individuals’ preferences for basic tastes^39–42^. There is evidence that trait openness to experience and extraversion correlate with particular food behavior. Conner et al.^43^, found that participants who scored above average on those traits, reported preference for salty, spicy and sour taste, and higher consumption of vegetables, fruits and healthy foods, as compared to less open, extraverted individuals. Also, food *neophilic* (novelty-seeker) and adventurous eaters tend to favor a wider range of nutrient-dense food and flavor combinations^44^, as opposed to anxious individuals (*neophobic*) reporting greater number of food aversions^45^. Studies conducted on sense of smell reported the modulatory effects of personality traits on olfactory perception—odor sensitivity, discrimination, identification –trait neuroticism one of the strongest predictors^45–49^. Specifically, it has been shown that highly anxious, neurotic individuals appear selectively biased towards affective odors, display higher olfactory acuity and reaction speed towards emotionally-valenced olfactory cues, and better performance on odors identification, discrimination as compared to less anxious, neurotic individuals^36,47,50^.

Further, previous research has also identified individual variations in the propensity to gather, utilize information through the haptic modality, namely the *Need for touch *^35^. Individuals high in *Need for touch* are more inclined to engage with haptic materials and tend to respond negatively when haptic exploration is impaired.

Taken together, these results point out the possible role of individual characteristics (haptic expertise, personality traits, Need for touch), alongside the properties of the material itself, in shaping haptic aesthetic appraisals. Yet our understanding of these putative factors remains limited. Existing research in this field is restricted in terms of the range of haptic stimuli explored, the kinds of sensory, and aesthetic responses that have been observed (apart from the like-dislike paradigm). Further, other crucial external factors have been often disregarded. For instance, the potential influence of context on haptic aesthetic appraisal. Indeed, the perceived pleasantness or unpleasantness of stimuli can vary depending on the context in which they are encountered: environmental temperature may affect perceptions of a copper plate’s coolness, making it haptically appealing during a torrid summer, yet unappealing in a rigid winter. Future investigations might want to take into account all these elements.

### The present research

In light of the extant body of research within the field of haptic aesthetics, the present study aims to more fully explore the haptic aesthetic appeal of visually presented materials surfaces, taking into account the impact of individual factors. To this end, a set of eighteen materials varying in typicality, and presented in the form of brief videos, were shown to a sample of participants varying in haptic expertise. Imagined perceptions of the material properties, complexity, familiarity, and haptic aesthetic responses were assessed via semantic differential scales. Individual differences in haptic expertise, personality traits and Need for touch were also measured. The study was designed to address the following research questions:

1. To what extent do materials’ sensory properties (e.g., smoothness vs. roughness) and individual subjective perceptions of complexity and familiarity influence the imagined haptic aesthetic appraisal of visually presented stimuli?
2. To what extent do individual differences in haptic expertise, Need for touch and personality traits influence the imagined haptic aesthetic appraisal of visually presented stimuli?

It was hypothesized that a material’s smoothness, slipperiness, and perceived familiarity would positively predict all facets of imagined haptic aesthetic appraisal in line with existing research conducted in uni-modal, bi-modal settings^21,22,24,32,51^. Moreover, it was predicted that a material’s perceived complexity would positively affect participants’ ratings of interest, in line with existing aesthetic theory^16^. Furthermore, given the top-down nature of haptic stimulus processing, and the presence of differences in motivational components relative to haptic exploration, it was hypothesized that individual differences—haptic expertise, Need for touch, openness to experience—would account for variations in levels of interest, and haptic invitation^35–38^. Specifically, high levels of haptic expertise, trait openness to experience and Need for touch were predicted to affect imagined haptic aesthetic ratings.

## Results

### Data preparation

First, we screened the data and excluded participants with large amounts of missing data, extremely short (< 720 s) or long completion times (> 7200 s) or responses polarized around the mean value on all semantic differential scales (SD < 1 around mean 3.8–4.2), leading to the exclusion of 188 participants. All variables were then inspected for univariate and multivariate outliers using *p* < 0.001 criterion for Mahala Nobis distance, and eleven participants were excluded on this basis, leaving a final sample of N = 347, representing a 36.45% loss of data, but exceeding the target sample size.

Results from assumptions of normality using the Shapiro–Wilk test, suggested several violations, nevertheless, all dimensions reported normal Q–Q plots of standardized residuals, and values for skewness and kurtosis within the acceptable limits, thereby no data transformations were applied. Residual and scatter plots indicated that assumptions of normality, homoscedasticity and linearity were all satisfied—few variables presented a high degree of association with each other. However, the Flat/Bumpy scale was excluded from further analysis due to its high level of collinearity with the Smooth/Rough scale^64^.

### Descriptive statistics

**Figure 1:**
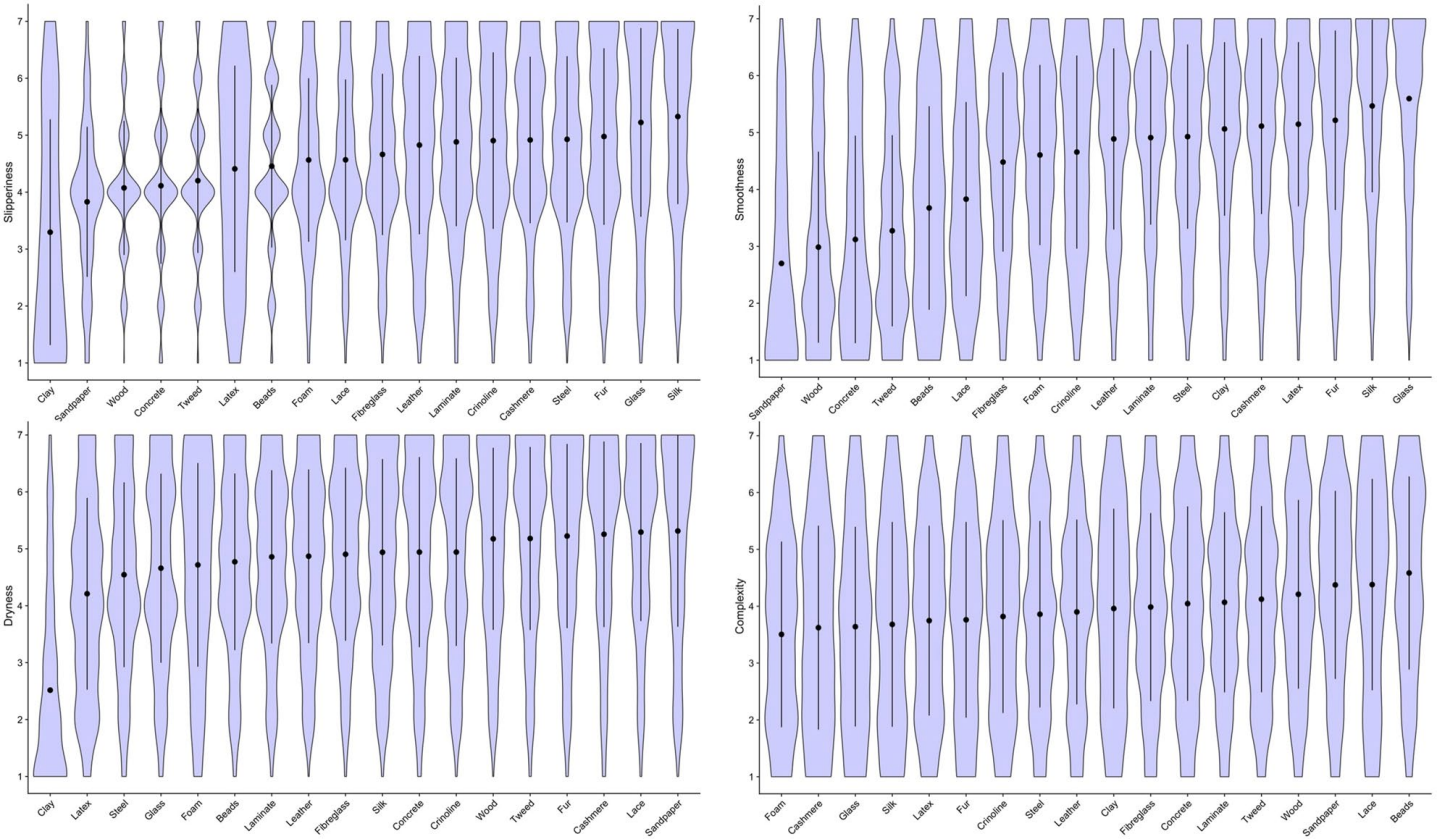
Violin plot overlaid with means and 95% CI for subjective ratings of perceived slipperiness (top left); smoothness (top right); dryness (bottom left); complexity (bottom right).

**Figure 2:**
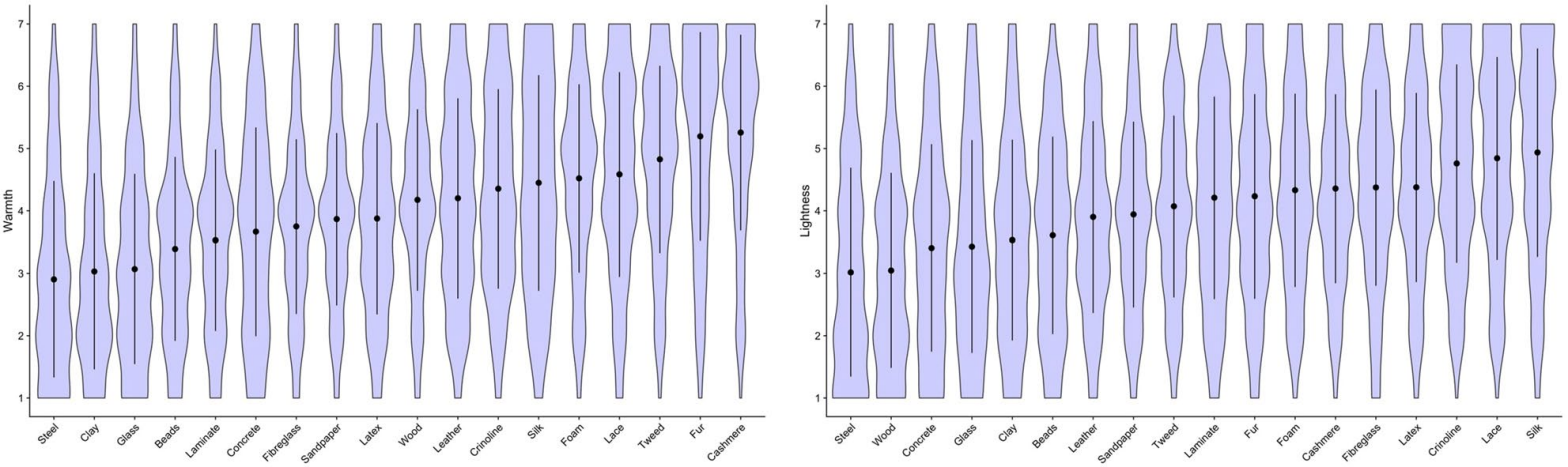
Violin plot overlaid with means and 95% CI for subjective ratings of perceived warmth (left); lightness (right).

**Figure 3:**
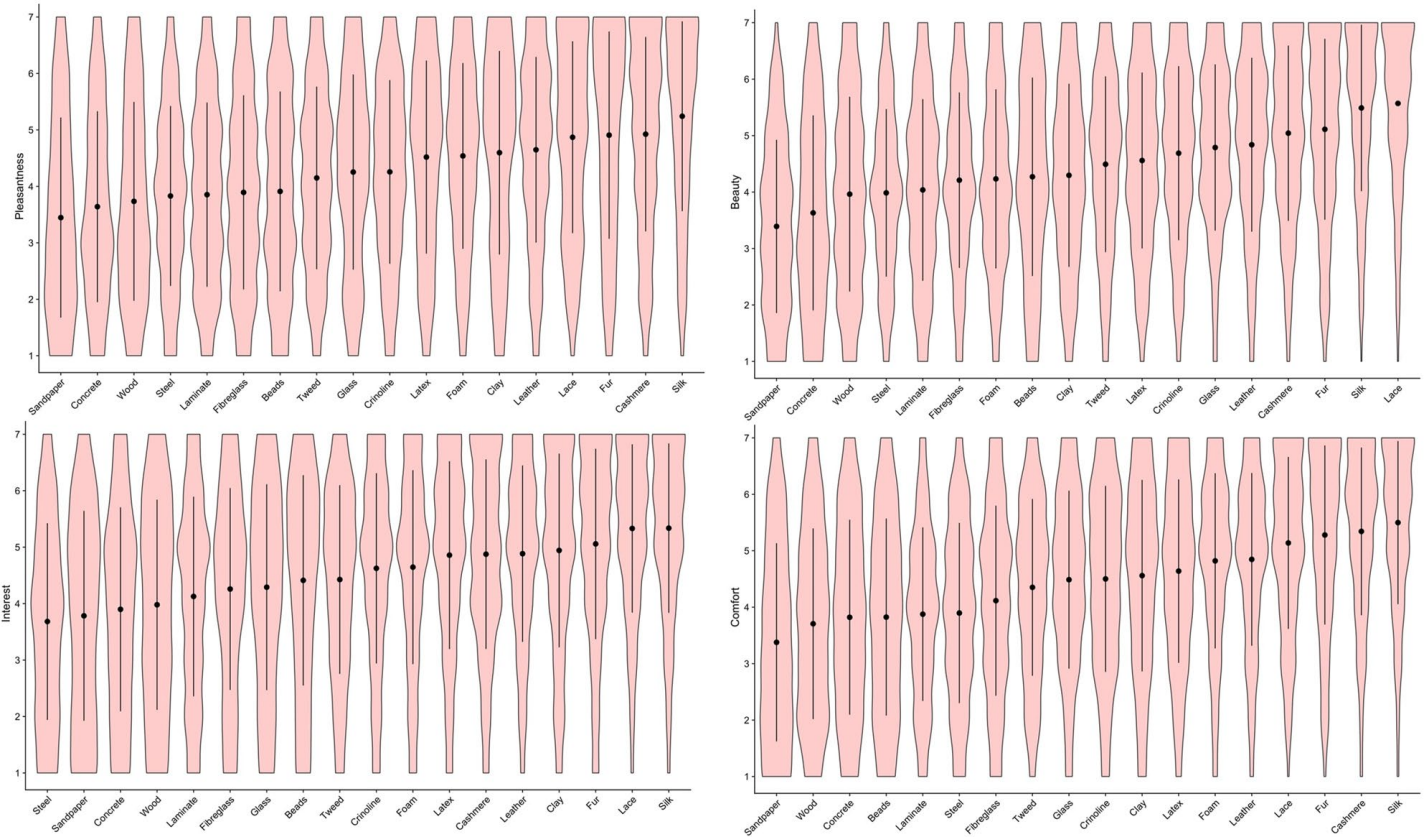
Violin plot overlaid with means and 95% CI for subjective ratings of perceived pleasantness (top left); beauty (top right); interest (bottom left); comfort (bottom right).

**Figure 4:**
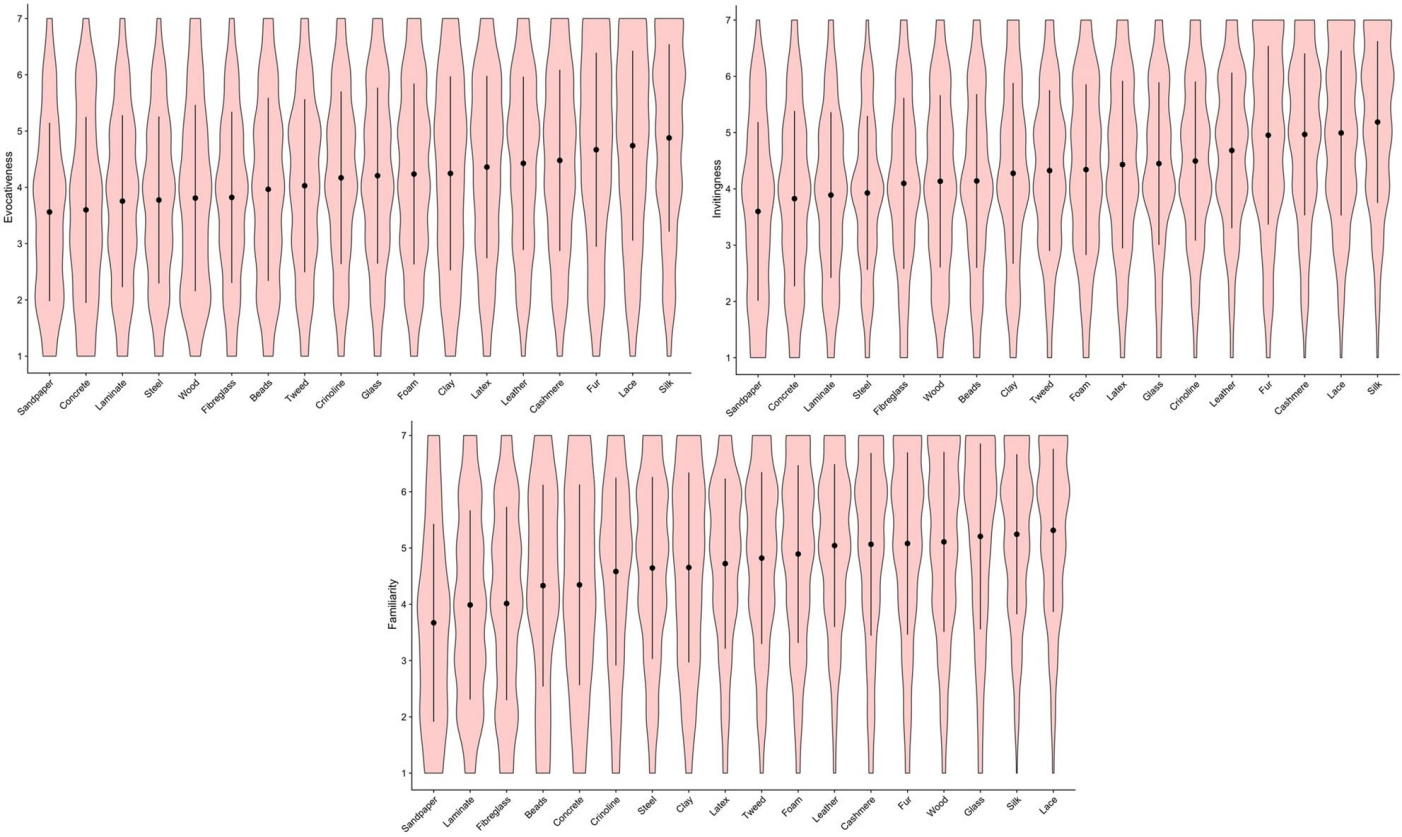
Violin plot overlaid with means and 95% CI for subjective ratings of perceived evocativeness (top left); invitingness (top right); familiarity (bottom left).

Means and standard deviations for haptic expertise, Need for touch and personality traits (Big Five Personality traits “BFI”) are given in Table 1. Figures 1 and 2 show the distribution of ratings for the perceived sensory properties of each material. As can be seen from these plots, glass, faux-fur and silk were judged as the smoothest and most slippery materials (Fig. 1). Dryness ratings for the materials showed relatively less variance, with clay being rated the least dry and sandpaper, lace and cashmere the driest (Fig. 1). Tweed, fur and cashmere were considered the warmest materials, with steel, clay and glass being the coolest. Crinoline, lace and silk were rated as the lightest materials, while steel and wood were rated as relatively heavier (Fig. 2). With regard to the haptically imagined aesthetic qualities of the materials, while there was some variability in how different stimuli were evaluated, sandpaper and concrete were consistently considered the least interesting, inviting, pleasant, evocative and beautiful (Figs. 3 and 4). On the other hand, silk, lace, fur and cashmere were consistently found to be the most interesting, inviting, pleasant, evocative, comfortable and beautiful relative to the other materials (Figs. 3 and 4).

**Table 1:** Means and standard deviations for haptic expertise, need for touch and BFI scores.

Individual difference measure		M	SD
BFI	Openness to experience	11.00	2.28
	Neuroticism	8.78	2.35
	Extraversion	9.84	2.21
	Agreeableness	10.40	1.87
	Conscientiousness	10.40	2.01
	Haptic expertise	28.10	16.90
	Need for touch	20.20	4.76

## Generalized linear mixed effects analyses

To investigate whether individual differences in personality traits, expertise and materials’ sensory properties predicted participants’ haptic aesthetic appeal of visually presented stimuli, we performed a series of generalized linear mixed effect analyses, modeling for variability at the stimulus and participant level. All analyses in the present research were conducted in R version 3.3.3. Significance was calculated using the lmerTest package^65^, which applies Satterthwaite’s method to estimate degrees of freedom and generates p-values for mixed models. The dependent measures of these analyses were subjective ratings of imagined haptic aesthetic appreciation: Interest, Pleasantness, Comfort, Invitingness, Beauty and Evocativeness. For all the analyses, subjects and materials were included as random intercepts.

### Linear mixed effects models of Sensory properties, Familiarity, and Complexity on Aesthetic responses

The maximum random effect’s structure justified by the data, contained random intercepts for participants and materials, but no random slopes:

$$ Interest \, / \, Pleasantness \, / \, Comfort \, / \, Beauty \, / \, Evocativeness \, / \, Invitingness \, \sim \, Familiarity \, / \, Complexity \, + \, Lightness \, + \, Warmth \, + \, Smoothness \, + \, Dryness \, + \, Slipperiness \, + \, \left( {1|subject} \right) \, + \, \left( {1|material} \right). $$

P-values were obtained by likelihood ratio tests of the full model with the effect in question against the model without the effect in question: *Interest/Pleasantness/Comfort/Beauty/Evocativeness/Invitingness* ~ *1* + (1*|Subject*) + (1*|Material*). Visual inspection of residual plots did not reveal any obvious deviations from homoscedasticity or normality.

The generalized linear mixed effect model for ratings of interest (Table 2) revealed positive effects of three sensory properties: perceived warmth, smoothness and lightness, and a negative effect of perceived dryness. Additionally, there was a significant positive effect of familiarity and complexity on interest ratings. Pleasantness and evocativeness ratings (Table 2 and 7), were significantly predicted by perceived warmth, smoothness, familiarity, lightness and slipperiness (Tables 3, 4, 5). Conversely, a reversed relationship was reported for perceived complexity, which was negatively related to pleasantness and evocativeness ratings. Finally, in the case of comfort, beauty and invitingness ratings (Tables 4, 6 and 7), the models showed significant positive effects of smoothness, familiarity, warmth, lightness and a negative effect of perceived dryness. Across all models, random effects for subjects were relatively large, compared with random effects for stimuli, meaning that there was a greater variance in response between participants than between different materials. In sum, the results suggest that the materials’ sensory properties: smoothness, warmth, lightness, slipperiness, alongside perceived level of familiarity and complexity have a positive impact on individuals imagined haptic appreciation.

**Table 2:** Generalized linear mixed effect model of material properties on interest ratings.

Interest: model fit			
Χ^2^(7) = 677.21**			

Fixed effects	Estimate	SE	t-value
Familiarity	0.12	0.01	9.41**
Complexity	0.06	0.01	4.89**
Lightness	0.11	0.01	8.26**
Warmth	0.18	0.01	13.08**
Smoothness	0.17	0.01	13.04**
Dryness	−0.05	0.01	−3.15**
Slipperiness	0.05	0.02	3.43**

Random effects	Variance	SD	
Intercept (subject)	0.50	0.71	
Intercept (material)	0.09	0.31	

In the table * indicates p < 0.05; ** indicates p < 0.001.

**Table 3:** Generalized linear mixed effect model of Material properties on Pleasantness ratings.

Pleasantness: model fit			
χ^2^(7) = 1010.90**			

Fixed effects	Estimate	SE	t-value
Familiarity	0.13	0.01	10.60**
Complexity	−0.07	0.01	−6.00**
Lightness	0.12	0.01	9.84**
Warmth	0.19	0.01	14.25**
Smoothness	0.15	0.01	12.45**
Dryness	−0.02	0.01	−1.43
Slipperiness	0.11	0.01	7.21**

Random effects	Variance	SD	
Intercept (subject)	0.38	0.62	
Intercept (material)	0.07	0.26	

In the table * indicates p < 0.05; ** indicates p < 0.001.

**Table 4:** Generalized linear mixed effect model of material properties on evocativeness ratings.

Evocativeness: model fit			
χ^2^(7) = 1007.40**			

Fixed effects	Estimate	SE	t-value
Familiarity	0.17	0.01	15.25**
Complexity	−0.07	0.01	−6.00**
Lightness	0.15	0.01	12.46**
Warmth	0.16	0.01	12.91**
Smoothness	0.07	0.01	6.05**
Dryness	−0.02	0.01	−1.60
Slipperiness	0.11	0.01	7.84**

Random effects	Variance	SD	
Intercept (subject)	0.30	0.55	
Intercept (material)	0.04	0.19	

In the table * indicates p < 0.05; ** indicates p < 0.001.

**Table 5:** Generalized linear mixed effect model of material properties on comfort ratings.

Comfort: model fit			
χ^2^(7) = 1213.80**			

Fixed effects	Estimate	SE	t-value
Familiarity	0.20	0.01	16.79**
Complexity	0.01	0.01	0.85
Lightness	0.08	0.01	6.40**
Warmth	0.17	0.01	13.34**
Smoothness	0.24	0.01	20.47**
Dryness	−0.10	0.01	−7.84**
Slipperiness	0.06	0.01	4.10**

Random effects	Variance	SD	
Intercept (subject)	0.18	0.43	
Intercept (material)	0.10	0.32	

In the table * indicates p < 0.05; ** indicates p < 0.001.

**Table 6:** Generalized linear mixed effect model of material properties on invitingness ratings.

Invitingness: model fit			
χ^2^(7) = 1091.70**			

Fixed effects	Estimate	SE	t-value
Familiarity	0.20	0.01	18.49**
Complexity	−0.06	0.01	−5.46**
Lightness	0.09	0.01	8.19**
Warmth	0.09	0.01	7.84**
Smoothness	0.20	0.01	17.84**
Dryness	−0.06	0.01	−4.99**
Slipperiness	< 0.01	0.01	0.23

Random effects	Variance	SD	
Intercept (subject)	0.19	0.44	
Intercept (material)	0.06	0.24	

In the table * indicates p < 0.05; ** indicates p < 0.001.

**Table 7:** Generalized linear mixed effect model of material properties on beauty rating.

Beauty: model fit			
χ^2^(7) = 1097.90**			

Fixed effects	Estimate	SE	t-value
Familiarity	0.21	0.01	18.59**
Complexity	−0.01	0.01	−1.05
Lightness	0.07	0.01	5.98**
Warmth	0.12	0.01	9.15**
Smoothness	0.22	0.01	18.99**
Dryness	−0.05	0.01	−3.93**
Slipperiness	0.05	0.01	3.41*

Random effects	Variance	SD	
Intercept (subject)	0.19	0.44	
Intercept (material)	0.13	0.36	

In the table * indicates p < 0.05; ** indicates p < 0.001.

### Linear mixed effects models of haptic expertise, need for touch and BFI personality traits on aesthetic responses

The maximal random-effects structure that converged included random intercepts for participants and materials, but no random slopes:

$$ Interest \, / \, Pleasantness \, / \, Comfort \, / \, Beauty \, / \, Evocativeness \, / \, Invitingness \, \sim \, Need \, for \, touch \, + \, Haptic \, Expertise \, + \, BFI \, Agreeableness \, + \, BFI \, Openness \, + \, BFI \, Conscientiousness \, + \, BFI \, Extraversion \, + \, BFI \, Neuroticism \, + \, \left( {1|subject} \right) \, + \, \left( {1|material} \right). $$

P-values were obtained by likelihood ratio tests of the full model with the effect in question against the model without the effect in question: *Interest/Pleasantness/Comfort/Beauty/Evocativeness/Familiarity/Invitingness* ~ *1* + (1|*Subject)* + (1*|Material*)*.* Visual inspection of residual plots did not reveal any obvious deviations from homoscedasticity or normality.

The generalized linear mixed effect model for ratings of interest (Table 8) revealed a significant positive effect of conscientiousness. Pleasantness ratings (Table 9) were significantly predicted by openness to experience and conscientiousness. In the case of comfort ratings (Table 10), the model revealed a significant positive effect of conscientiousness, and negative effects of trait Openness to experience and Neuroticism. For invitingness ratings (Table 11), the fit of the overall model in relation to the null was non-significant. Beauty ratings (Table 12) were positively predicted by conscientiousness and Neuroticism. Finally, evocativeness ratings (Table 13) were positively predicted by openness to experience and conscientiousness. Across all models, random effects for both subjects and materials were relatively large.

**Table 8:** Generalized linear mixed effect model of personality traits on Interest ratings.

Interest: model fit			
χ^2^(7) = 18.42*			

Fixed effects	Estimate	SE	t-value
Need for touch	< 0.01	0.01	0.40
Haptic expertise	< 0.001	< 0.01	−1.66
BFI agreeableness	< 0.01	0.03	−0.31
BFI openness	0.03	0.02	1.31
BFI conscientiousness	0.07	0.03	2.71*
BFI extraversion	−0.04	0.02	−1.72
BFI neuroticism	−0.03	0.02	−1.26

Random effects	Variance	SD	
Intercept (subject)	0.55	0.74	
Intercept (material)	0.24	0.49	

In the table * indicates p < 0.05; ** indicates p < 0.001.

**Table 9:** Generalized linear mixed effect model of personality traits on pleasantness ratings.

Pleasantness: model fit			
χ^2^(7) = 59.38**			

Fixed effects	Estimate	SE	t-value
Need for touch	< 0.01	< 0.01	0.43
Haptic expertise	< 0.01	< 0.01	−0.62
BFI agreeableness	0.02	0.03	0.59
BFI openness	0.08	0.02	3.38**
BFI conscientiousness	0.10	0.03	4.01**
BFI extraversion	< 0.01	0.02	0.38
BFI neuroticism	< 0.01	0.02	−0.03

Random effects	Variance	SD	
Intercept (subject)	0.47	0.68	
Intercept (material)	0.25	0.50	

In the table * indicates p < 0.05; ** indicates p < 0.001.

**Table 10:** Generalized linear mixed effect model of personality traits on comfort ratings.

Comfort: model fit			
χ^2^(7) = 36.22**			

Fixed effects	Estimate	SE	t-value
Need for touch	< 0.01	< 0.01	0.88
Haptic expertise	< 0.01	< 0.01	1.71
BFI agreeableness	−0.03	0.02	−1.55
BFI openness	−0.04	0.02	−2.01*
BFI conscientiousness	0.06	0.02	2.76*
BFI extraversion	< 0.01	0.02	0.15
BFI neuroticism	−0.05	0.02	−3.53**

Random effects	Variance	SD	
Intercept (subject)	0.27	0.52	
Intercept (material)	0.37	0.60	

In the table * indicates p < 0.05; ** indicates p < 0.001.

**Table 11:** Generalized linear mixed effect model of personality traits on invitingness ratings.

Invitingness: model fit			
χ^2^(7) = 9.93			

Fixed effects	Estimate	SE	t-value
Need for touch	0.01	< 0.01	2.03
Haptic expertise	< 0.01	< 0.01	0.03
BFI agreeableness	−0.02	< 0.01	−0.82
BFI openness	0.01	0.02	−0.55
BFI conscientiousness	0.03	0.02	1.60
BFI extraversion	−0.03	0.02	−1.70
BFI neuroticism	−0.02	0.02	−1.15

Random effects	Variance	SD	
Intercept (subject)	0.29	0.54	
Intercept (material)	0.19	0.43	

In the table * indicates p < 0.05; ** indicates p < 0.001.

**Table 12:** Generalized linear mixed effect model of personality traits on beauty ratings.

Beauty: model fit			
χ^2^(7) = 14.66*			

Fixed effects	Estimate	SE	t-value
Need for touch	0.01	< 0.01	1.71
Haptic expertise	< 0.01	< 0.01	-0.31
BFI agreeableness	< 0.01	0.02	-0.22
BFI openness	-0.01	0.02	-0.56
BFI conscientiousness	0.04	0.02	2.10*
BFI extraversion	-0.02	0.02	-1.13
BFI neuroticism	-0.04	0.02	-2.22*

Random effects	Variance	SD	
Intercept (subject)	0.30	0.55	
Intercept (material)	0.33	0.58	

In the table * indicates p < 0.05; ** indicates p < 0.001.

**Table 13:** Generalized linear mixed effect model of personality traits on evocativeness ratings.

Evocativeness: model fit			
χ^2^(7) = 53.84**			

Fixed effects	Estimate	SE	t-value
Need for touch	< 0.01	< 0.01	0.95
Haptic expertise	< 0.01	< 0.01	0.34
BFI agreeableness	0.02	0.02	0.75
BFI openness	0.08	0.02	3.67**
BFI conscientiousness	0.07	0.02	3.10*
BFI extraversion	< 0.01	0.02	0.36
BFI neuroticism	0.02	0.02	0.86

Random effects	Variance	SD	
Intercept (subject)	0.42	0.64	
Intercept (material)	0.14	0.38	

In the table * indicates p < 0.05; ** indicates p < 0.001.

## Discussion

The primary purpose of the current study was to examine the influence of materials’ sensory properties and individual differences on the haptic aesthetic appeal of visually presented stimuli. Considering the ratings provided by participants on the imagined perception of material properties, we found that silk, glass, faux-fur were considered as the smoothest and most slippery materials, while sandpaper was rated as the driest, roughest and least slippery. These findings align with existing research adopting both haptic unimodal, and bimodal visuo-tactile conditions^20,21,32,51^. Furthermore, aesthetic responses marked out silk, lace and fur as the most preferred stimuli in terms of beauty, comfort and interest ratings, while sandpaper and concrete were often rated the lowest in these dimensions.

Linear mixed effects analyses revealed consistent and significant associations between imagined material properties and aesthetic responses, as well as between individual differences and aesthetic responses, although to a weaker degree. Across the models tested we saw a positive effect of familiarity, warmth, smoothness, and lightness on aesthetic responses to the materials, supporting existing research and theory that suggests that the apparent safety, familiarity and comfort of a material drives haptic aesthetic responses to that material^32^. In particular, the perception of a given material’s warmth may have potentially triggered implicit memories of trust, comfort, or prenatal experience within the maternal womb—given the early ontogeny of the haptic modality^2,3,66–68^. Preference for safe, familiar stimuli may be an innate propensity or the result of acquired knowledge and experience^69^, highlighting a role for both implicit, explicit memories in individuals’ aesthetic judgments of haptic materials, and the top-down nature of haptic aesthetic appreciation. The current study is unable to speak directly to the question of at what stage such preferences for haptic stimuli emerge, and developmental research would be a valuable way to further explicate the mechanisms underlying these preferences.

In the current study, complexity ratings for the materials positively predicted interest ratings but negatively predicted pleasantness, invitingness, and evocativeness ratings. This aligns with the findings of Berlyne^16^ in the visual, auditory domains in which complexity differentially predicted interest and aesthetic pleasure based on mechanisms of arousal. Interest, and pleasure are generally described as distinct routes to liking, pleasure being a hedonic response in automatic processing, whereas interest is linked to an increase in the desire to explore stimuli^15^. Given that haptic exploration not only stems from autotelic but also from discovery purposes, a certain degree of complexity might be crucial in providing an optimal level of arousal in haptic experience. However, the present study did not aim to explicitly address participants’ aesthetic responses towards materials that were systematically varied in terms of their complexity. Additionally, the measurement of complexity was only derived subjectively rather than considering the objective complexity of each stimulus. Future research may consider investigating whether different levels of complexity produce variation in participants’ perceived interest and pleasantness, and whether that interacts with relevant individual differences (e.g., trait curiosity and openness to experience).

Turning to the analysis of individual differences in relation to imagined haptic aesthetic appreciation, personality traits have been previously shown to affect the way sensory, emotional/affective information is processed across domains^36–38^. This appears to be the case in the present study, where openness to experience, neuroticism, and conscientiousness predicted facets of imagined haptic aesthetic appreciation. Specifically, openness to experience and conscientiousness positively predicted pleasantness and evocativeness ratings, while openness to experience negatively predicted comfort ratings. Additionally, conscientiousness positively predicted interest and comfort ratings. Much research has indicated that openness to experience relates to aesthetic sensitivity^70^. Highly open individuals display a more positive aesthetic attitude and increased receptivity to sensory stimuli^71,72^. This theoretical account potentially explains the observed positive relationship emerged with ratings of pleasantness and evocativeness. Nevertheless, it is unclear why trait openness to experience failed to significantly positively correlate with all other facets of haptic aesthetic appreciation. The fact that participants could not explore the materials using the haptic modality in the current study may have introduced a source of bias in the interpretation of the adjectives used to aesthetically evaluate the materials, which then potentially interacted with some of the personality traits explored in the current study.

Amongst all big five personality traits investigated in the present study, conscientiousness appeared as the strongest predictor of facets of haptic aesthetic appreciation. Trait conscientiousness refers to an individual’s level of persistence, organization, dependability, self-discipline and goal-oriented behaviors. Within aesthetic research, conscientiousness has often displayed weak or null patterns of association with aesthetic experiences^73–76^. A possible reason that we observed an effect of conscientiousness in the current study may be the phenomenon of “extreme response style”. In questionnaire-based studies, and in the presence of high levels of extraversion and conscientiousness, it was observed that participants are keen to favor extreme response categories, thereby resulting in possible correlation between ratings^49,77–79^.

Haptic expertise did not significantly predict any of the measures of haptic aesthetic appreciation in the current study, contrary to predictions drawn from the expertise literature in other aesthetic domains. The way in which haptic expertise was measured may have been too liberal in terms of the specific demands of the current task, which was to evaluate the sensory and aesthetic properties of textile materials using vision alone. We may yet discover differences between those with and without haptic expertise in the context of a study where participants are required to explore the materials using haptic exploration. More generally, it is important to note that the generalized mixed effect analyses conducted on individual differences reported poorer model fit as compared to those on sensory properties, suggesting that individual differences played a less prominent role in determining haptic aesthetic appreciation compared with the perceived sensory properties of the materials.

There are limitations of the current research. Data collection restrictions imposed by the COVID-19 global pandemic led us to adopt a visual unimodal, rather than bimodal, which required participants to judge the haptic properties form the materials using vision alone, potentially leading to biases in the aesthetic and sensory ratings provided. It would be therefore advisable to conduct a similar experiment in person with unimodal (vision only) bimodal (vision and touch) conditions, to assess whether the current results match those under conditions including haptic exploration. However, the current results do have interesting implications in the context of digital advertising (e.g., fashion, product/interior design) in which potential consumers are required to evaluate the sensory and aesthetic properties of materials without being able to touch them.

To conclude, the present research aimed to advance theoretical understanding of the relationship between material properties and haptic aesthetic appreciation, taking into account individual differences in personality traits and haptic expertise. This study contributes to the current knowledge of haptic aesthetics and paves the way for future investigations which can probe in depth the role of different sensory modalities and individual differences on our aesthetic experience of haptic exploration. This research also points to interpersonal differences as potential factors that might be considered into a variety of product domains, (industrial, product, interior, fashion and architectural design) to better target and fulfill consumers’ haptic needs.

## Methods

### Participants

A total number of 546 participants (293 female, 14 “other”, M_age_ = 27.8 years, SD_age_ = 6.26, range = 19–60 years) took part in the study on a voluntary basis. Prior to participant recruitment, a power analysis was conducted for sample size estimation. Sample size estimation of Linear Mixed Effects Models (LMEMs) is notoriously challenging as it requires specifying reliable beta coefficients, fixed effects and variance of random effects (Kumle et al. 2021). Due to the absence of prior research on the effect of stimulus properties on haptic aesthetics, insufficient existing data were therefore available to simulate the LMEMs used in the current study. Therefore, it was deemed appropriate to conduct, a sample size estimation, using G* Power, for multiple regression with power (1 − β) set at 0.80, adjusted α = 0.01 based on the number of hypotheses tested, d = 0.15. This yielded a total sample of N = 323 people to detect small effects, with power of 0.80.

Participants were recruited in two steps: via online sampling, targeting diverse web sources (e.g., *Reddit, Survey-circle, Gumtree*); and via purposive sampling, from national and international universities (e.g., *Goldsmiths University of London, Parsons school of design-The new school*). Recruitment from specialist university programs took into account the amount, frequency of manual activities involved in the study pathways: only programs requiring high levels of manual dexterity, and haptic exploration/manipulation as their “core” activity on a weekly basis were considered. All participants were fluent in English, reported normal or corrected to normal vision, and gave their informed consent before taking part in the experiment. The present study received ethical approval from the Research Ethics Committee at Goldsmiths, University of London, and has been performed in accordance with the Declaration of Helsinki.

## Materials

### Stimuli

A set of eighteen material surfaces varying in typicality was used in the experiment, with *silk* and *sandpaper* being the most common, and *crinoline* and *fiberglass* the most unusual. The stimuli were presented in an indirect manner (visual information only), given constraints in in-person data collection related to the COVID-19 pandemic. The stimuli were therefore presented in the form of brief videos, lasting for approximately 10 s, which were accompanied by a label displaying the texture’s name and showed a human hand exploring the material surface with different strategies (Exploratory Procedures).

Three different *Exploratory Procedures* (*EPs*) were employed in line with Lederman and Klatzky's^52^ theoretical framework on haptic perception, to promote a more accurate representation of the material properties and also to evoke real life haptic explorations in the participants: a “*lateral motion” EP,* linked to the perception of texture (e.g., roughness); *“static contact” EP*, for the assessment of thermal properties; and *“unsupported holding” EP*, associated with the material’s weight. The integration of refined *EPs* and the presence of a human hand touching the material were used to enhance the tactile properties of the materials given that they were presented in the visual modality only (via activation of the somatosensory cortex^53–56^). The sound produced by the stroking of the actor’s skin on the material surface was removed, to avoid potential confounding effects given by cross-modal interactions.

**Figure 5:**
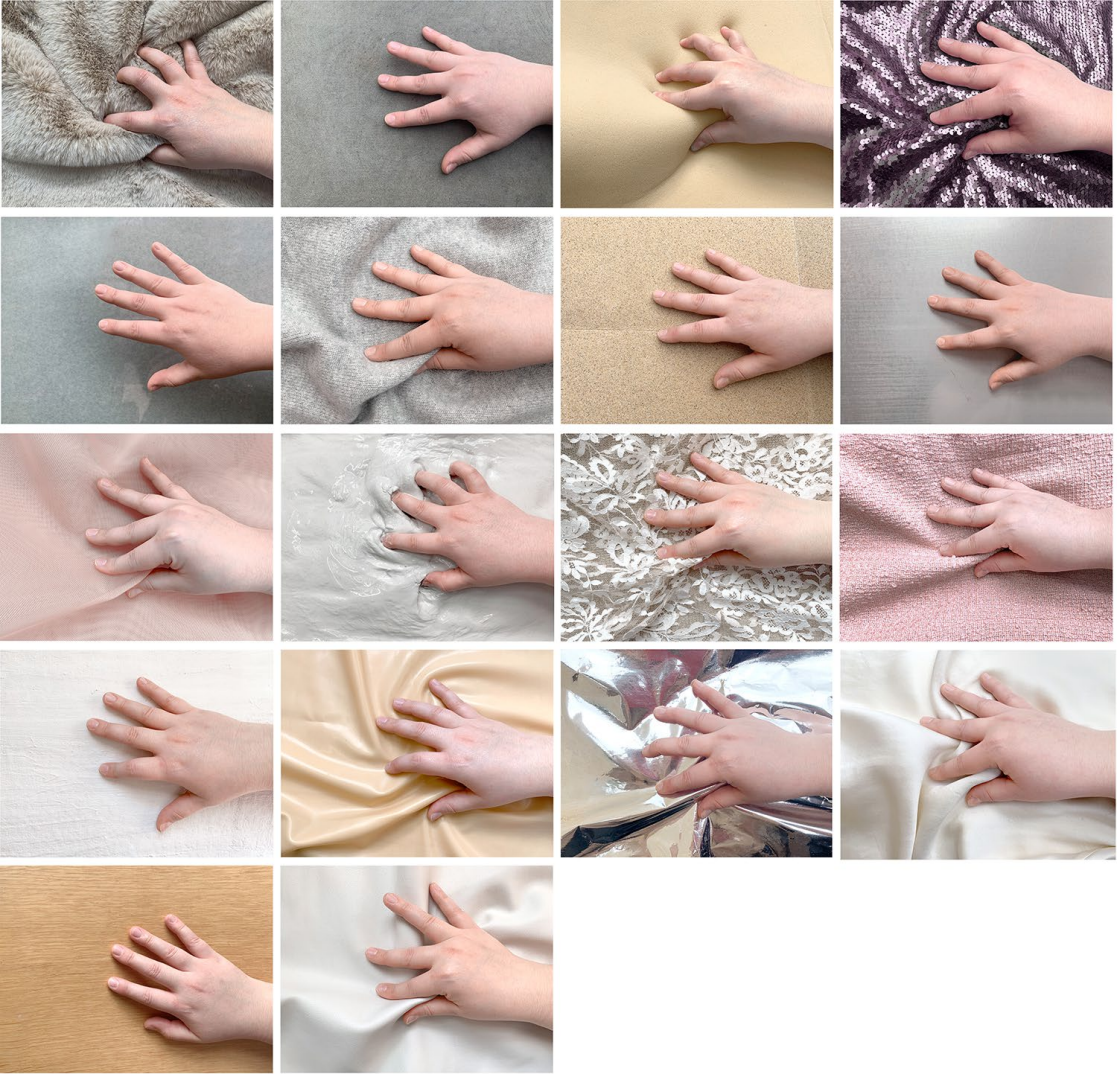
Final set of stimuli. The presented pictures represent an extract from the 10 s videos with the actor’s hand manipulating the material surfaces. From the top left moving clockwise: 1, faux-fur; 2, concrete; 3, low-density foam; 4, beads; 5, glass; 6, cashmere; 7, sandpapers; 8, steel; 9, crinoline; 10, clay; 11, lace; 12, tweed; 13, fiberglass; 14, latex; 15, laminate; 16, silk; 17, wood; 18, leather.

The materials were selected drawing from the existing body of research in haptic aesthetics^20,20,20,21,51,57^, and various artistic, non-artistic domains; subsequently, they were evaluated and reduced by fifteen experts in artistic and manual fields with 10+ years of experience (e.g., sculptors, fashion designers) during a pilot study. Neutral, pale shades (white, skin-tones, sand, light gray) were favored over vivid ones and gray scale, to minimize potential confounding effects and to preserve textures’ definition. Materials were also screened for: lack of clarity, prototypicality, and undesirable similarity to other items, yielding a total number of eighteen final stimuli (Fig. 5).

### Measures

To investigate participants’ imagined perceptions of sensory, aesthetic qualities of the materials, as well as their perceived familiarity and complexity, a 7-point semantic differential scale was administered, including fourteen antonym pairs of adjectives. The semantic differential scales were drawn from the Sensory Emotional scale of Touch perception^58^, and the existing haptic literature exploring touch perception and surface properties^21,34,59,60^. This yielded six items related to aesthetic appreciation, six items related to the material’s sensory properties, and two additional items to assess perceived familiarity and complexity of the material. Words on each semantic differential scale were randomly polarized and presented in a random order. The antonym pairs related to the perceived sensory properties of the material were: smooth/rough; warm/cold; dry/wet; flat/bumpy; slippery/sticky and light/heavy. Antonym pairs related to the aesthetic qualities of the material were: interesting/boring; pleasant/unpleasant; comfortable/uncomfortable; beautiful/ugly; inviting/repelling; evocative/not evocative. Additional antonym pairs relating to complexity and familiarity were: complex/simple; familiar/unfamiliar.

Individual differences were assessed focusing on three dimensions: the participant’s experience with tasks that require high haptic ability (expertise) and haptic efficiency (manual dexterity); the natural tendency to engage in haptic explorations for aesthetic purposes (Need for touch); and personality traits. To capture each participants’ haptic expertise and manual dexterity, a compacted and revisited version of the Touch Experience Questionnaire developed by Guest et al.^58^ was used, in which participants reported musical instruments and type of music they played, artistic experiences, hobbies involving touch, and the extent to which their job-domain required haptic ability. Ratings were made via selecting which of the five levels of experience (ranging from 5 = expert, and 0 = No experience) described the participant’s level of familiarity or skills for the items in the scale sections. To identify individual differences in participants’ motivation to touch objects or materials for hedonic purposes, known as Need for touch, the Need for touch (NFT) scale developed by Peck and Childers^35^ was employed. However, for the present study purposes, only six items pertaining to the Autotelic NFT subscale were used, measured on an adjusted 5-point Likert scale (anchored to 1 = Strongly disagree, and 5 = Strongly agree). Finally, participants’ personality traits were measured using The Big Five short inventory questionnaire (BFI)^61^, consisting of fifteen items, measured on a 5-point Likert scale (1 = Strongly disagree, 5 = Strongly agree).

### Procedure

The experiment was conducted remotely, using the online software platform Qualtrics—each participant could partake in the study using a laptop or tablet. Participation was on a voluntary basis. All data, including age, gender, nationality and language spoken were recorded through the platform software. The experiment consisted of three blocks of six video trials, each block followed by a questionnaire. There was no time limit for completion of the study. The whole procedure lasted approximately 35 min per participant.

In each trial participants were presented with a video showing a human hand manipulating a material, lasting for approximately 10 s. If required, participants could see the video repeatedly. Alongside the video, a 7-point semantic differential scale with fourteen items was displayed. Participants were instructed to imagine exploring the material’s surface with their hands, and then rate it on the list of attributes provided (see materials). In situations where touch is not available, giving participants the instruction to “mentally explore” the material, may promote volitional mental imagery of touch, further strengthening “a simulation of touch”, already elicited by the sight of a touch percept^55,56,62,63^. At the end of Block 1 participants completed the Touch Experience questionnaire^25^, in Block 2 they completed the BFI short inventory^61^ and in Block 3 they completed the Need for touch Questionnaire^35^.

## Data Availability

The datasets generated and analyzed during the current study are available in the Open Science repository, https://osf.io/3bsvp/?view_only=d86ba57142fc4b8289c62901356f6fdf.
